# A Nationwide Survey on Patient’s versus Physician´s Evaluation of Biological Therapy in Rheumatoid Arthritis in Relation to Disease Activity and Route of Administration: The Be-Raise Study

**DOI:** 10.1371/journal.pone.0166607

**Published:** 2016-11-28

**Authors:** Sophie De Mits, Jan Lenaerts, Bert Vander Cruyssen, Herman Mielants, René Westhovens, Patrick Durez, Dirk Elewaut, on behalf of the Be-Raise study group

**Affiliations:** 1 Department of Rheumatology, Ghent University Hospital, Ghent, Belgium; 2 Department of Rheumatology, Reuma-Instituut Hasselt, Leuven University Hospital, Leuven, Belgium; 3 Department of Rheumatology, Sint Jozef Hospital Bornem, OLV Hospital Aalst, Aalst, Belgium; 4 Skeletal Biology and Engineering Research Center, Department of Development and Regeneration KU Leuven; Rheumatology, University Hospitals Leuven, Leuven, Belgium; 5 Department of Rheumatology, Université Catholique de Louvain UCL, Louvain-la-Neuve, Belgium; 6 Laboratory of Molecular Immunology and Inflammation, VIB Inflammation Research Center, Ghent University, Ghent, Belgium; VU University Medical Center, NETHERLANDS

## Abstract

**Objectives:**

Biological treatment of rheumatoid arthritis (RA) is one of the cornerstones of current treatment strategies for the disease. Surprisingly little information exists on whether the route of administration affects patients’ treatment satisfaction. It is equally unclear whether rheumatologists are able to accurately perceive their patients’ appreciation. Thus, the Belgian Be-raise survey aimed to examine whether RA patient’s experience of their current biological treatment coincided with the treating physician’s perception.

**Methods:**

A nationwide cross-sectional survey was conducted by 67 Belgian rheumatologists providing data obtained from 550 RA patients. Patients under stable dose of biologics for at least 6 months, were enrolled consecutively and all completed questionnaires. Separate questionnaires were completed by the treating rheumatologist which evaluated their patient’s perception of the route of treatment administration. This study therefore evaluates whether a treating physician perceives the satisfaction with the route of administration to the same degree as the patient.

**Results:**

Completed questionnaires were obtained from 293 and 257 patients who obtained treatment via the intravenous (IV) or subcutaneous (SC) route of administration, respectively. 58.4% of patients were in DAS28-CRP(3) remission. Patient satisfaction with disease control was higher (44% scored ≥ 9) than that of the treating physician (35%), regardless of the route of administration (p< 0.01). No differences were seen for the patients treated with an IV as opposed to a SC route of administration. The physician´s perception of patient’s satisfaction with disease control was markedly lower for IV treated patients as opposed to SC treated patients (p< 0.001).

**Conclusions:**

Patients’ satisfaction with biological treatment is high, but there is a considerable mismatch between patients´ and rheumatologists´ appreciation on the route of administration of biological therapy in RA. Physicians consistently consider IV biological therapy to be less satisfactory. Patient´s appreciation is largely dependent on disease control, irrespective of the route of administration. Therefore, and encouraging shared decision making, we suggest that physicians and patients discuss the route of administration of biologicals in an open way.

## Introduction

Over the past two decades the introduction of biologics has caused a paradigm shift in the treatment of diseases such as rheumatoid arthritis (RA). The efficacy of these biologicals has been evaluated in clinical trials using classical clinical outcome measures such as ACR20, HAQ, X-ray scores and DAS28 [1, 2]. Incorporating outcome measures such as DAS scores, imaging, as well as an evaluation of the patient’s quality of life has changed treatment targets in clinical practice and improved patient outcome.

In recent years the development of pre-filled syringes has promoted subcutaneous (SC) administration of biologicals and has therefore become an attractive alternative to an intravenous (IV) route of administration [3]. In this context, several studies have investigated the efficacy and safety of different biological therapies available in either IV or SC formula (eg. abatacept, golimumab, adalimumab, tocilizumab) and found no difference between these modes of administration [2, 4–6].

Although the route and frequency of administration might affect the adherence to treatment [7, 8], to date only a limited number of studies have examined the patients’ preference for one route of administration above another [7–12]. The majority of patients appear to prefer a SC route of administration for biologicals [8–10, 12], with only one study showing no preference between either IV or SC administration [7]. In this context, factors favoring IV infusion of biologicals in patients include: the safety of hospital administration and the presence of medical staff, social contact in the hospital, patient compliance and the possibility for regular checkups. Factors supporting a SC route on the other hand include: the comfort and convenience of treatment at home, avoiding/minimizing time of transportation for treatment, improving the quality of life due to less interference with the everyday life and the feeling of greater independence, freedom and the capacity for self-management [1, 5, 7, 8, 11, 13]. Age, frequency of administration, re-imbursement for one admission mode versus the other and confidence in the treating physician decision might also be of influence for the patient preferences for either the IV or SC route of administration [7, 8, 14–17].

Although the treatment choice should preferably be a shared patient–physician decision, as stated in the Eular recommendations [18], the physician’s judgment and the patient’s trust in the physician seem to have a greater effect on the confidence in the treatment decision rather than the patient’s knowledge of potential treatment options [16, 18, 19]. However, involvement in the decision-making process might enhance patient’s satisfaction with and adherence to therapy [10].

Since the physician’s opinion plays an important role in the patient choice of route of administration, it seems important to know if the patient and the physician would make the same choice if they make this independently from one another. Thus far, to our knowledge, only two studies have questioned health professionals about their preference for one route of administration above another (ie. IV vs SC). In one study physicians were asked to complete a questionnaire as if they were patients themselves [7]. In contrast, Willeke et al. conducted a survey of RA patients’ and of rheumatologists’ preferences for IV or SC administration. However, the rheumatologists were not the treating physicians of the patients participating in the study [20].

Thus, we propose that both patient and treating physician alike may benefit from a study which investigates how each party experiences the treatment of RA. Factors that are of interest here include the general satisfaction of disease control as well as the convenience of the route of administration of current biologics.

We therefore initiated a nationwide survey called Be-Raise (Belgian Rheumatoid Arthritis: Patient Insights, Strategies and Expectations), which aimed to investigate how patients and their treating physicians experience treatment of RA. In addition, this study investigated if patient’s and physician’s perception was similar.

## Materials and Methods

Be-Raise is a nationwide cross-sectional survey, which invited all Belgian rheumatologists to participate. RA patients under a stable biological therapy for at least 6 months were selected consecutively in an outpatient clinic or day-hospital. This selection was made to avoid patients that were still in a phase of therapy adjustment with a biologic. In all participating practices, each rheumatologist could include a maximum of 10 RA patients. The study was approved by the Ethical Committee of Ghent University Hospital.

Both patients and physicians completed a similar questionnaire, newly constructed for this purpose. The difference in the questionnaires can be seen in the phrasing: the patient’s version uses the words ‘you’ and ‘yours’, whereas the physician’s version uses the words ‘your patient’. Furthermore, the patient’s version started with collecting data about age, sex, date of diagnosis, employment and marital status. The physicians were asked about their years of experience as a rheumatologist and about their clinical setting (academic/peripheral/private practice, rheuma nurse availability). The physicians also provided some extra patient information about the current disease activity (CRP, ESR, global VAS, joint score).

Since SC treated patients have a different participation in their therapy compared to IV treated patients, the phrasing of the questionnaires was adapted to the route of administration. The patient’s questionnaires pertaining to SC or IV administration differed only in the questions concerning administration. More specifically, patients receiving biologics intravenously were asked to score the treatment within a hospital setting whereas patients administered biologics subcutaneously were asked to score for more variables. The SC patient had to choose between the section ‘you are self-administering your biological treatment’ or ‘your biological treatment is given at home by someone else than yourself’ and received additional questions pertaining to drug preparation and storage. Table 1 summarizes what was asked of patients and physicians in the questionnaires. The full version of the questionnaires can be found in the supplementary material (S1–S3 Questionnaires).

**Table 1:** The different items covered by the questionnaires.

The patient’s questionnaire
• demographic data
• medication use: biological and concurrent medication for rheumatoid arthritis
• how the choice for biological treatment was made
• the evaluation and satisfaction of the effect of their biological treatment on symptoms and daily live activities
• the practical aspects of their treatment and the satisfaction with the way of administration
• therapy compliance
• the knowledge about and attitude towards possible side effects during treatment with biologicals
• the preference of administration (regardless of their current form of therapy)
The physician’s questionnaire
• information about the physician’s experience and clinical setting
• information about the patient: disease duration and disease activity, medication
• how the choice for the biological treatment was made
• the evaluation and satisfaction of the effect of the patient’s biological treatment on symptoms and daily live activities
• the practical aspects of the patient’s treatment and his/her satisfaction with the way of administration
• the patient’s therapy compliance
• the patient’s perception about the safety of the use of biologicals

Patients and treating physicians completed questionnaires independently of one another and in a blinded fashion. At the central collection point, the coded questionnaires were merged and a supplemental control of matching was performed by verifying sex and age.

Most questions about satisfaction were reported on a 0–10 Likert scale. Good satisfaction was defined by a score of ≥ 9.

### Statistics

Descriptive analyses were included in the calculations of proportions for categorical variables and means/medians with 95% confidence intervals.

DAS28 was calculated according to the DAS28-CRP(3) formula. DAS28-CRP(3) was used as the standard covariate in regression analyses, but all regression analyses were double checked with the DAS28-ESR(3). Generalized linear model analysis was used with a logit link for dichotomous data and an identity link for continuous data. Missing data were handled by the ‘missingness completely at random’ (MCAR) assumption [21]. Propensity scores were calculated as described by Joffe & Rosenbaum [22].

All statistics were calculated with PASW 18 “SPSS 18” (Chicago, Illinois, US).

## Results

Of the 200 registered Belgian rheumatologists, 67 (30%) of those working in 37 private practices and outpatient settings of hospitals participated in this study. Questionnaires were obtained from 550 patients of which 293 received drugs IV and 257 SC. Physician-patient matched questionnaires were obtained for 260 IV and 231 SC treated patients, respectively. Missing value analyses did not suggest against the MCAR assumption. While we have not recorded the information of non-included patients in all centers, data from one center (Ghent University Hospital) would not suggest that this is the case as included patients did not differ in all characteristics (such as DAS28) compared to non-included patients.

The demographic and baseline characteristics of patients and the treatment they received are shown in Table 2. There were no differences between the IV group and the SC group. Patients (n = 550) were treated with Etanercept (17.1%), Adalimumab (25.6%), Certolizumab (0.9%) Golimumab (2.7%), Rituximab (10.0%), Abatacept (8.7%), Infliximab (20.9%) or Tocilizumab (14%).

**Table 2:** Demographic and baseline characteristics.

	all	IV	SC
**age (years)**	57.7 ± 12.34	57.7 ± 12.16	57.8 ± 12.56
**gender**			
male (n)	114 (20.7%)	63 (23.2%)	51 (21.3%)
female (n)	389 (70.7%)	205 (75.4%)	184 (76.7%)
missing (n)	47 (8.5%)		
**disease duration (years)**	3.3 ± 0.81	3.4 ± 0.80	3.3 ± 0.83
**clinic type**			
university hospital (n)	199 (36.2%)	129 (47.54%)	65 (27.1%)
private hospital (n)	188 (34.2%)	92 (33.8%)	74 (30.8%)
private outpatient clinic (n)	133 (24.2%)	34 (12.5%)	88 (36.7%)
missing (n)	30 (5.2%)	17 (6.2%)	13 (5.5%)
**CRP (mg/l)**	12.4 ± 55.83	14.1 ± 74.7	10.3 ± 29.02
**ESR (mm/h)**	15.0 ±18.51	16.4 ± 16.7	13.2 ± 19.93
**global VAS (mm)**	23.7 ±20.77	21.6 ± 19.3	25.5 ± 22.02
**DAS-28-3 CRP**	2.6 ±1.06	2.5 ± 1.02	2.6 ± 1.11
**remission (n)**	321 (58.4%)	167 (61.4%)	138 (57.5%)

IV: intravenous, SC: subcutaneous, CRP: C-reactive protein, ESR: erythrocyte sedimentation rate

Further analysis was performed only on matched (physician–patient) questionnaires.

### Patient’s and physician’s satisfaction with control of RA symptoms

**Fig 1:**
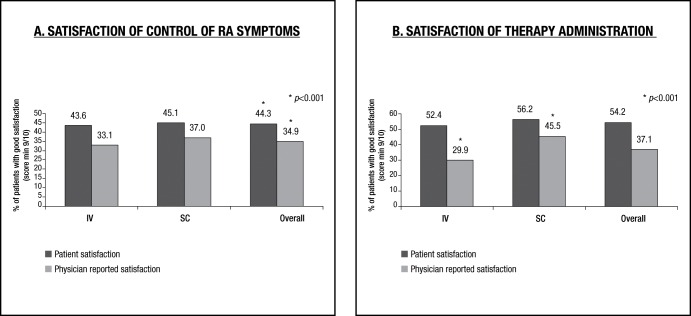
Patient satisfaction and associated physician’s opinion of control of RA symptoms and on therapy administration. IV: Intravenous; SC: subcutaneous.

The patient’s satisfaction on the overall control of RA symptoms was higher than the treating physician’s, regardless of the route of administration. 44% of patients were satisfied (score ≥ 9) about symptom control, compared to 35% of the treating physicians (OR = 3.9 (2.6–5.8) p < 0.001) (Fig 1 panel A). Furthermore, no differences were seen for the patients treated with an IV compared to a SC route of administration.

### Patient’s and physician’s satisfaction with the route of therapy administration (IV vs SC)

Conversely, the physician´s perception of patient’s satisfaction with disease control was noticeably lower for IV treated patients as opposed to SC treated patients. Here, 70% of physicians estimated that IV treated patients were less satisfied with an IV route of administration (Fig 1 panel B) as opposed to 54% for SC treated patients (OR = 1.9 (1.4–2.8) p< 0.001).

Further logistic regression analyses, corrected for a propensity score for IV/SC allocation (including sex, age, professional status and disease duration etc.) revealed that patients are significantly more satisfied with the route of administration when they are also comfortable about disease control (OR = 4.1, 95% CI 2.7–6.2 p<0.0001). In contrast, neither the route of administration (OR = 0.77, 95% CI 0.46–1.27, p = NS) nor the frequency of administration (OR = 0.71,95%CI 0.47–1.07, p = NS) affected the appreciation of therapy administration.

Finally, the physician’s preferred therapy administration differs from that of the patient’s. IV use was consistently rated lower than SC use (OR = 0.531, 95% CI = 0.302–0.934, p = 0.028), irrespective of disease activity or frequency of therapy administration. Different sensitivity analyses with continuous data (generalized linear modeling with identity link) and alternative DAS formulas confirmed these findings.

## Discussion

The results from this nationwide study highlight that in approximately 50% of the cases recorded, patients´ and rheumatologists´ judgement differ in the context of disease control and the degree of satisfaction related to the route of administration of biological therapy in RA. Physicians consistently consider IV biological therapy to be less satisfactory, whereas the patient´s appreciation is independent of the route of administration. This study underlines the finding that the primary factor promoting patient satisfaction is good disease control.

The finding that 52.4% of patients were satisfied with an IV route of administration as opposed to 56.2% of patients receiving SC administration is in accordance with previous studies which show a preference for SC over IV administration of biologicals [8–10, 12].

Although age has been shown to influence a preference for IV above SC drug administration in patients [7, 8, 17], within our patient population there was no significant difference (p = 0.70) between the mean age for the IV or SC group, being 57.7 ± 12.16 years and 57.8 ± 12.56 years, respectively.

Distance and time to treatment and re-imbursement have been named as possible factors influencing the choice of treatment [7, 8, 11, 17]. Within Belgium’s social and economic context, this might be of minor importance. It is a small country with good accessibility to different hospitals and out-patient clinics and the patient is free to choose his specialist. Biological therapy can only be prescribed by a rheumatologist, guided by restricted rules concerning reimbursement. However, these rules are the same for the different biological therapies and for the different routes of administration, and are therefore of no influence in the decision on IV or SC administration [7–10, 12, 17].

Although our questionnaire did not explicitly address whether physicians preferred one route of drug administration above another, we assume they rank oral administration above SC and IV administration, respectively. However, for a patient with a chronic potentially disabling disease, it would appear that the route of administration is of less importance to the patient as long as the treatment is effective. Thus, encouraging shared decision making, we suggest the physicians to discuss this matter in an open way.

The findings of this study are in agreement with results presented by Willeke et al. where it is suggested that rheumatologists overestimate the preference of patients for SC injections. Willeke et al. revealed 88% of physicians preferring the SC mode and 92% of the physicians assumed that patients would do so as well, while 92% of the patients would choose again for intermittent IV therapy [20]. In this study it was shown that 52,4% of patients were satisfied with an IV route of administration whereas only 29.9% of their treating physicians thought they were pleased with the IV route of administration.

To our knowledge, this study is the first to compare patients’ and physicians’ evaluation on the route of administration. This large nationwide dataset, allowing adjustments with regression analysis, and the blinded comparison of patients’ and physicians’ opinion on a large set of questions, reinforces the findings of this study. Nevertheless, this study is limited by the cross sectional design and these results have to be interpreted within Belgium’s social and economic context.

Currently, patient participation in treatment decision making is actively encouraged. It is also known that a patient’s trust in his/her physician has a significant effect on the confidence in the type of treatment prescribed. Thus, future studies should address the degree of discrepancy between the rheumatologist’s and patient’s perception, preference and expectations for a specific treatment regime. Furthermore, how the frequency of drug administration affects the choice of treatment also requires further study.

## Conclusion

In conclusion, this study demonstrates patient’s preference and satisfaction for a biological treatment to be independent of the route of administration, but primarily to be driven by the patient’s satisfaction about the treatment effectiveness. In contrast, physicians consistently consider IV biological therapy to be less satisfactory for patients.

## Supporting Information

S1 Questionnaire Be-Raise Questionnaire patient_IV. (PDF)

S2 Questionnaire Be-Raise Questionnaire patient_SC. (PDF)

S3 Questionnaire Be-Raise Questionnaire physician. (PDF)
