# Supplementary material for: A Nationwide Survey on Patient’s versus Physician´s Evaluation of Biological Therapy in Rheumatoid Arthritis in Relation to Disease Activity and Route of Administration: The Be-Raise Study
Source: PLoS One. 2016 Nov 28;11(11):e0166607. doi: 10.1371/journal.pone.0166607 (PMC5125609; doi:10.1371/journal.pone.0166607)
Supplement: S2 Questionnaire — (PDF) [file pone.0166607.s002.pdf]

Patient Questionnaire SC

Patient's code number 01/01/01

Your participation in this survey consists in completing this questionnaire carefully, fold and mail it without a stamp (postage is at our expense) or deliver it in a sealed envelope to your doctor. Please do not write down your name and address on the envelope and on this questionnaire.

An amount of 10 € by completed questionnaire will be paid to the "Fund for Scientific Research in Rheumatology". In this way you bring your support to scientific research on rheumatoid arthritis, which ultimately benefits all patients who suffer from it.

Thank you in advance for your cooperation!

Are you ? ☐ man ☐ woman How old are you ? |__|__| years

When were you diagnosed with rheumatoid arthritis?
☐ since less than 1 year ☐ between 1 and 5 years ☐ between 5 and 10 years ☐ more than 10 years

What is your employment status?
☐ active full-time ☐ active part-time because of illness ☐ active part-time
☐ liberal professional / independent ☐ retired ☐ job seeker
☐ student ☐ on mutual (disability / incapacity)
☐ other: ________________________________

What is your marital status? ☐ married ☐ single ☐ cohabiting

YOUR TREATMENT

1. a- What biological treatment are you currently taking?

☐ Enbrel ☐ Humira ☐ Mabthera ☐ Orencia ☐ Remicade ☐ RoActemra

b- How long do you take this biological treatment?

Since | _____ | months/years *(please circle the appropriate time unit)*

c- Before the current biological treatment, have you taken other biological treatments?
☐ no

☐ yes → which one before the current one? |__________________| time: ______ Weeks/Months/Years

→ which ones before that, if any? |__________________| time: ______ Weeks/Months/Years

Other, please specify ....

The questions below relate to the drug that you checked above (a).

2. a- Did you know, before they were prescribed by your doctor, the "biologic therapies" for treating rheumatoid arthritis (RA)?

☐ yes → go to question 2b

☐ no → go to question 3

b- How did you hear about these drugs? *(Please circle the appropriate number(s)*- multiple answers possible)

Medical staff	1
Family members / friends with RA	2
Family members / friends without RA	3
Media (TV, newspapers, radio, support group, literature)	4
Internet	5
Patient association	6
Other, more precisely............................	7

3. Is there a reuma- nurse in the medical service/cabinet?

☐ no ☐ yes ☐ do not know

If "yes":

☐ Were you visited by the rheuma-nurse? ☐ no ☐ yes

☐ Did you receive any information (eg. about administration, … of the current biological treatment) from this rheuma-nurse? ☐ no ☐ yes

4. Besides this biological treatment, what other treatments are you currently receiving for your rheumatoid arthritis?

Ledertrexate ☐ Dose: ______ mg/ week → Tablets ☐ Injection ☐

Arava ☐ Dose: ______ mg/day

Salazopyrine ☐ Dose: ______ mg/day

Plaquenil ☐ Dose: ______ mg/day

Cortisone ☐ Dose: ______

Anti-inflammatory drugs (eg. Brufen, Voltaren,...) : ___________ ☐ Dose: ______

Other: ______________ ☐ Dose: ______

CHOICE OF BIOLOGICAL TREATMENT

5. How was the final choice of your biological treatment made?

❑ My physician has described the possible treatments and I have chosen together with the physician which treatment I was going to follow (--> the physicians suggested different treatments and the patient chooses)

❑ My physician explained which treatment was best for me and I'm following his recommendations (--> the physician decides)

❑ I have discussed with my physician a treatment for which I had done some research. The physician prescribed me this treatment. (--> the physician prescribes what the patient suggests)

6. What were the ultimately decisive arguments for the choice of biological treatment with which you are being treated?

(Please circle the appropriate number(s)- multiple answers possible)

What I could expect from the medicine (efficacy, effect on symptoms ...)	1
How the medicine works	2
The differences between the mode of action of the different biological treatments	3
How the medicine must be administered	4
How often the medicine must be administered	5
Who must administer the medicine	6
Where the medicine is administered (hospital vs home)	7
The possible side effects at the time of administration	8
Potential risks of the use of this medicine in the long term	9
Other factors, please specify :……………………………………………………………………	10

7. To which extend the doctor (or other medical staff, where appropriate) has informed you about the following elements with regard to biological treatment in general or specifically on the biological treatment prescribed to you?

Please fill in a figure between 1 and 10 for each of the statements, whereby:

1	2	3	4	5	6	7	8	9	10
Not at all informed									Very well informed

What I could expect from the medicine (efficacy, effect on symptoms …)	|____|
How the medicine works	|____|
The differences between the mode of action of the different biological treatments	|____|
How the medicine must be administered	|____|
How often the medicine must be administered	|____|
Who must administer the medicine	|____|
Where the medicine is administered (hospital vs home)	|____|
The possible side effects at the time of administration	|____|
Potential risks of the use of this medicine in the long term	|____|

Other elements, please specify…….

EVALUATION OF YOUR BIOLOGICAL TREATMENT

8. a- To what extent are you satisfied about the effectiveness of the biological treatment in terms of **relief/control of your RA symptoms**? *Please circle the level of satisfaction below.*

1	*2*	*3*	*4*	*5*	*6*	*7*	*8*	*9*	*10*
Not at all satisfied								*Very satisfied*	

b- To what extent are you satisfied about the effect of the biological treatment on the **possibility to perform your daily activities and enjoy life**? *Please circle the level of satisfaction below.*

1	*2*	*3*	*4*	*5*	*6*	*7*	*8*	*9*	*10*
Not at all satisfied								*Very satisfied*	

c- Could you indicate to what extent you agree with the following statements about your medication? Please indicate in the box a number from 1 to 4 that corresponds to your opinion.

1 ❑ not agree at all   3 ❑ agree
2 ❑ not really agree   4 ❑ totally agree

a- my treatment prevents or delays the progression of joint damage	| ____ |
b- my treatment prevents or delays the progression of my RA	| ____ |
c- my treatment gives a short-term relief of my symptoms	| ____ |
d- my treatment gives a long-lasting relief from my symptoms	| ____ |
e- my treatment decreases the number of flares of my RA	| ____ |
f- my treatment is the newest, most advanced	| ____ |
g- my treatment remains effective between two administrations	| ____ |
h- my treatment is an easy treatment	| ____ |
i- my treatment gives irritation (redness / itching) at injection site	| ____ |
j- my treatment causes pain at the moment of injection	| ____ |

THE PRACTICAL ASPECTS OF THE TREATMENT

9. a- To what extent are you satisfied about the method of administration of your biological treatment (taking into account all aspects of **administration** such as method, who and where the treatment is administered)?
Please circle the figure corresponding to the satisfaction below.

1 2 3 4 5 6 7 8 9 10
Not at all satisfied *Very satisfied*

b- Why are you giving this score?
____

____

10. Where and by whom is your biological treatment administered?

☐ at home by yourself (go to question 11)
☐ at home by someone else → by whom?
(go to question 12)
☐ partner ☐ nurse
☐ generalist ☐ other : --
→ what is the reason why you do not administer your biological treatment yourself?
☐ fear of self-injection
☐ not able to, my hands do not allow me
☐ not able to so because of other physical reasons
☐ other: -- --------------------

IF YOU ARE SELF-ADMINISTERING YOUR BIOLOGICAL TREATMENT

11. a- Here is a series of statements relating to the administration of your biological treatment. To what extent do you agree with each statement? *Please enter a number from 1 to 10 for each of these aspects*

1 2 3 4 5 6 7 8 9 10
Totally disagree *Totally agree*

a- It was clearly explained to me how to administer the product |____|

b- Opening the package is easy |____|

c- The instructions for use are clear and structured |____|

d- Administering the injection is easy |____|

e- I may experience pain when injecting it |____|

b- Where do you inject the biological drug? (*Several answers possible*)

❑ Arm ❑ Thigh ❑ Belly ❑ other location: where ? ______________________

c- Are there other advantages or disadvantages to the administration of your treatment that you find important?
____
____

IF YOUR BIOLOGICAL TREATMENT IS GIVEN AT HOME BY SOMEONE ELSE THAN YOURSELF:

12. a-Here is a series of statements relating to the administration of your biological treatment by the person administering the medication. To what extent do you agree with each statement? *Please enter a number from 1 to 10 for each of these aspects.*

1 2 3 4 5 6 7 8 9 10
Totally disagree *Totally agree*

a- Opening the package seems easy for him / her | ____ |

b-The instructions for the person administering the biological treatment | ____ |

seems clear and structured | ____ |

c-The administration of the injection is easy, goes well | ____ |

-d Pain may occur during injection | ____ |

b- Where is the injection of the biological drug done? (*Several answers possible*)

❑ Arm ❑ Thigh ❑ Belly ❑ other location: where ? ______________________

c- Are there other advantages or disadvantages to the administration of your treatment that you find important?

____

____

THERAPY COMPLIANCE

13. a- How often does the administration of your biological treatment takes place too late (i.e. not on the scheduled day)? *Please fill in a figure between 1 and 10 for each of these aspects.*

1 2 3 4 5 6 7 8 9 10
Never Not often Regularly Very often

b- Which of the following reasons have been a reason to postpone or skip the administration of the treatment at a certain time? *Several answers possible*

☐ difficult to administer, too much hassle ☐
☐ fear for pain and discomforts during injection ☐
☐ fear for injection site reaction ☐
☐ help required from caretaker ☐
☐ I feel well enough to skip doses or to leave more time between 2 administrations ☐
☐ too much other medication which I also have to remember to take ☐
☐ I am worried about the long-term effects ☐
☐ on holiday or another change in daily routine ☐
☐ insufficient result to be worth it ☐
☐ do not understand the instructions very well ☐
☐want as little medication as possible in my body ☐
☐ price of the treatment ☐
☐ scheduled surgery ☐
☐ infection, flu, fever, … ☐
☐ simply forgot, no specific reason ☐
☐ other, please specify: _________________ ☐

c-What do you do when you have forgotten your administration?

☐ I contact my general practioner

☐ I contact the rheumatologist

☐ I contact the nurse

☐ I administer as quickly as possible the injection

☐ I do nothing and wait time of next administration

☐ Other:

SECTION ON THE CONSERVATION OF BIOLOGICAL DRUG

14. a-If you or someone else bought your biological drug at the pharmacy …

- Are you going straight home mostly/ the person who bought it, bring it directly to your home?
☐ yes ☐ no

- Are you / the person who bought it, using a cool bag to keep the drug at recommended temperature for the transport to your home?
☐ yes ☐ no

b-At what temperature do you keep the biological drug at home …?
☐ at room temperature ☐ in the fridge

c-Are you carrying your biological drug while traveling …?
☐ simply in a travel bag ☐ in a special cool bag

SECTION ABOUT THE CHOICE BETWEEN THE DIFFERENT OPTIONS OF ADMINISTRATION

15. Please specify, for each of the following proposals, which of the two or three suggestions you prefer, regardless of treatment you are currently receiving. *Only one answer per line possible!*

☐ conventional injection or ☐ automatic injection system (pen) or ☐infusion

☐ Monthly injections at home or ☐ 1 infusion at the hospital every 2 months

☐ less frequent administrations or ☐ fewer side effects

☐ Fewer side effects or ☐ good control of disease activity

☐ Monthly injection at home or ☐ one infusion in the hospital every 6 months

☐ personal preference of administration mode or ☐ good control of disease activity

POTENTIAL SIDE EFFECTS DURING TREATMENT WITH BIOLOGICAL DRUG

16. What was /or is your attitude if you experience side effects during or immediately after the administration of your medication? *One possible answer!*

☐ take necessary action as I was told

☐ contact my general practitioner

☐ contact the rheumatologist

☐ contact the nurse

☐ contact another physician, namely: ______________________________

☐ ask advice to the pharmacist

☐ I do nothing and wait until it passes

17. To what extent do you think your biological treatment is safe?
Please circle the number that corresponds to your opinion

1	*2*	*3*	*4*	*5*	*6*	*7*	*8*	*9*	*10*
Not safe at all									*very safe*

18. a-During the prescription of the treatment did someone informed you about the risk of certain side effects ?

☐ no
☐ yes → Who provides you this information?
☐ the general practioner
☐ rheumatologist
☐ nurse at the hospital
☐ nurse at home
☐ the pharmacist
☐ the patient organization
☐ other person, specifically: ___________________________________

b-Would you like to receive more information on possible side effects that may occur during (or immediately after) the administration of your medication?
☐ no
☐ yes → By whom this information should be given preferably?
Several answers are possible, please indicate the importance with a number in the right column.

☐ the general practioner	☐
☐ rheumatologist	☐
☐ nurse at the hospital	☐
☐ nurse at home	☐
☐ the pharmacist	☐
☐ the patient organization	☐
☐ other person, specifically: ___________________________________	☐

c-Have you received / searched information about potential safety risks via other sources?

☐ no
☐ yes → Via what other sources?

☐ Family ☐ Internet ☐ Patients brochures ☐ Pharmacist
☐ Patient organization ☐ other: ____

d- Here is a series of statements related to precaution of use of biological treatments for RA. Could you indicate whether you think these statements are "true" or "false"?

- In case of flu symptoms biological treatment can be continued

☐ true ☐false

- A patient who receives biological treatment can be vaccinated against influenza

☐ true ☐ false

- Biological treatment increases the risk of respiratory infections

☐ true ☐ false

- In rare cases cancer can occur

☐ true ☐ false

- Tuberculosis can occur with biological treatment

☐ true ☐ false

- For traveling to the tropics, I have to seek additional advice

☐ true ☐ false

- Biological treatment can cause malformation to unborn children

☐ true ☐ false ☐ do not know

- Biological treatment can be given to patients with heart failure

☐ true ☐ false ☐ do not know

- Pregnant women can continue to take their biological treatment

☐ true ☐ false ☐ do not know

19. What has been / or would be your attitude if you were / are facing a specific problem such as fever or other special situation? *Only one answer possible!*

☐ take necessary action myself as I was told

☐ contact my general practitioner

☐ contact the rheumatologist

☐ contact the nurse

☐ contact another doctor, namely: ______________________________

☐ ask advice to the pharmacist

☐ I do nothing and wait until it passes

Thank you for your participation in this survey!
