# Supplementary material for: A Nationwide Survey on Patient’s versus Physician´s Evaluation of Biological Therapy in Rheumatoid Arthritis in Relation to Disease Activity and Route of Administration: The Be-Raise Study
Source: PLoS One. 2016 Nov 28;11(11):e0166607. doi: 10.1371/journal.pone.0166607 (PMC5125609; doi:10.1371/journal.pone.0166607)
Supplement: S3 Questionnaire — (PDF) [file pone.0166607.s003.pdf]

Rheumatologist Questionnaire

Physician number 01/01/01 **Patient's sex: ☐ M ☐ F** **Patient's age: ____ yrs.**

Physician's information

How long have you been an accredited rheumatologist: ____ yrs.

The patient is being treated at an
☐ Academic institution
☐ Peripheral institution
☐ Private practice

Is a reuma- nurse available at the institution/practice?

☐ no

☐ yes → was the patient visited by this reuma- nurse? ☐ no ☐ yes

→ was information (with regard to administration, ... of the current biological treatment) given? ☐ no ☐ yes

Patient information

1. RA diagnosis since ____/_____/_____

2. a- Which biological treatment is the patient currently taking?

☐ Enbrel ☐ Humira ☐ Mabthera ☐ Orencia ☐ Remicade ☐ RoActemra

b- How long has he or she been taking this biological treatment?

Since |_____| months/years *(please circle the appropriate time unit)*

c- Was he or she taking other biological treatments before the current biological treatment?

☐ no

☐ yes → which one before the current one? |___________________| time: ______ Weeks/Months/Years

→ which ones before that, if any? |___________________| time: ______ Weeks/Months/Years

Other, please specify ....

3. Besides this biological treatment, which other treatments is your patient currently receiving for his or her RA?

Ledertrexate ☐ Dose: ______ mg/week → Tablets ☐ Injection ☐
Arava ☐ Dose: ______ mg/day
Salazopyrine ☐ Dose: ______ mg/day
Plaquenil ☐ Dose: ______ mg/day
Cortisone ☐ Dose: ______
Anti-inflammatory agents (like Brufen, Voltaren, ...): _____________ ☐ Dose: ______
Others: _____________ ☐ Dose: ______

CHOICE OF BIOLOGICAL TREATMENT

4. How was the final choice of biological treatment made for this patient?

❑ I have discussed the possible treatments and I chose the treatment he or she was going to follow together with the patient (--> you suggested different treatments and the patient chooses)

❑ I explained which treatment was best for the patient and he or she is following this treatment because I suggested it (--> I decide)

❑ The patient looked up the information and I prescribed the medication to him or her (--> I prescribe what the patient suggested)

5. To which extent does the patient think that you, the doctor (or other medical staff, where appropriate) has informed him or her about the following elements with regard to the biological treatment?

Please fill in a figure between 1 and 10 for each of the statements, whereby:

1 2 3 4 5 6 7 8 9 10
Not at all informed ... *Very well informed*

What he could expect from the medicine (efficacy, effect on symptoms …)	|____|
How the medicine works	|____|
The differences between the action of the different biological treatments	|____|
How the medicine must be administered	|____|
How often the medicine must be administered	|____|
Who must administer the medicine	|____|
Where the medicine is administered (hospital vs home)	|____|
The possible side effects at the time of administration	|____|
Potential risks of the use of this medicine in the long term	|____|

EVALUATION OF HIS BIOLOGICAL TREATMENT

6. a- To what extent do you think your patient is satisfied about the effectiveness of the biological treatment in terms of **relief/control of his or her RA symptoms**?
Please circle the level of satisfaction below.

1 2 3 4 5 6 7 8 9 10
Not at all satisfied ... *Very satisfied*

b- To what extent do you think your patient is satisfied about the effect of the biological treatment on the **possibility to perform his or her daily activities and enjoy life**?
Please circle the level of satisfaction below.

1 2 3 4 5 6 7 8 9 10
Not at all satisfied ... *Very satisfied*

THE PRACTICAL ASPECTS OF THE TREATMENT

7. To what extent do you think **your patient is satisfied about the method of administration** of his or her biological treatment (taking into account all aspects of administration such as method, who and where the treatment is administered)? *Please circle the figure corresponding to the satisfaction below.*

1 2 3 4 5 6 7 8 9 10
Not at all satisfied ... *Very satisfied*

IF THE BIOLOGICAL TREATMENT IS ADMINISTERED AT THE HOSPITAL

8. How do you think your patient answered the questions below?

Please fill in a figure between 1 and 10 for each of these aspects.

1	2	3	4	5	6	7	8	9	10
Totally disagree									*Totally agree*

a- My treatment is adequately monitored by the physician/nurse |____|

b- The duration of the administration is acceptable in terms of length of time |____|

c- The period between two administrations is acceptable |____|

d- The administration at the hospital puts me at ease, gives confidence |____|

e- My treatment is/was clearly explained |____|

IF YOUR PATIENT ADMINISTERS THE BIOLOGICAL TREATMENT HIMSELF OR HERSELF

9. How do you think your patient answered the questions below?

Please fill in a figure between 1 and 10 for each of these aspects.

1	2	3	4	5	6	7	8	9	10
Totally disagree									*Totally agree*

a- It was clearly explained to me how to administer the product |____|

b- Opening the package is easy |____|

c- The instructions for use are clear and structured |____|

d- Administering the injection is easy |____|

e- I may experience pain when injecting it |____|

THERAPY COMPLIANCE

10. a- How often do you think the administration of his or her biological treatment takes place <u>too late</u> (i.e. not on the scheduled day)?

Please fill in a figure between 1 and 10 for each of these aspects.

1	2	3	4	5	6	7	8	9	10
Never		*Not often*				*Regularly*			*Very often*

b- Which of the following reasons have been a reason for the patient to postpone or skip the administration of the treatment at a certain time? *Several answers possible*

- ❑ difficult to administer, too much hassle
- ❑ fear for problems caused by injection / fear for pain and discomforts when placing the infusion
- ❑ fear for reaction to the injection / infusion reactions
- ❑ help required from caretaker
- ❑ patient feels well enough to skip doses or to leave more time between 2 administrations
- ❑ too much other medication which he or she also has to remember to take
- ❑ is worried about the long-term effects
- ❑ on holiday or another change in daily routine
- ❑ insufficient result to be worth it
- ❑ does not understand the instructions very well
- ❑ wants as little medication as possible in his or her body
- ❑ price of the treatment
- ❑ simply forgot, no specific reason
- ❑ scheduled surgery
- ❑ infection, flu, fever, ...
- ❑ other, please specify: _________________

THE SAFETY OF THE USE OF BIOLOGICAL TREATMENTS

11. What does your patient think about the safety of the biological treatment?
Please circle the figure corresponding to his or her opinion below.

**1**	**2**	**3**	**4**	**5**	**6**	**7**	**8**	**9**	**10**
Not at all safe									*Very safe*

CURRENT DISEASE-ACTIVITY

12. Patient's clinical evaluation

CRP: _______ mg/dl ESR: _______ mm/h

13. Physician's GLOBAL VAS

0 1 2 3 4 5 6 7 8 9 10

← Low activity level High activity level →

14. Evaluation of the 28 joints

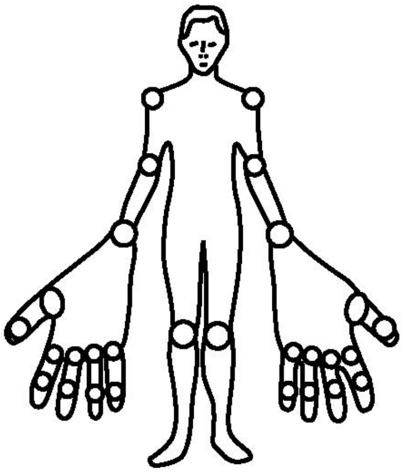

/___ ___/
Number of painful joints

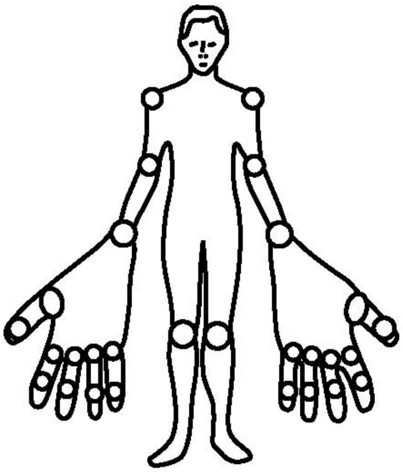

/___ ___/
Number of swollen joints

Thank you for completing this survey!
